# Disentangling perceptual judgment and online feedback deficits in Parkinson’s freezing of gait

**DOI:** 10.1007/s00415-015-7759-7

**Published:** 2015-05-01

**Authors:** Carolina R. A. Silveira, Kaylena A. Ehgoetz Martens, Frederico Pieruccini-Faria, Danielle Bell-Boucher, Eric A. Roy, Quincy J. Almeida

**Affiliations:** Sun Life Financial Movement Disorders Research and Rehabilitation Centre, Wilfrid Laurier University, 75 University Avenue West, Waterloo, ON N2L 3C5 Canada; Department of Kinesiology, University of Waterloo, Waterloo, ON Canada; Department of Psychology, University of Waterloo, Waterloo, ON Canada; Department of Psychology, Wilfrid Laurier University, Waterloo, ON Canada; Department of Health and Rehabilitation Sciences, Western University, London, ON Canada

**Keywords:** Parkinson’s disease, Freezing of gait, Gait, Space perception, Sensory feedback, Planning

## Abstract

Although the underlying mechanisms of freezing of gait in Parkinson’s disease (PD) are not fully understood, impaired sensory–perceptual processing has been proposed as an important contributor to freezing episodes. The aims of this cross-sectional study were to disentangle how sensory–perceptual deficits involved in planning (prior to movement) and sensory–perceptual feedback processing (during movement execution) contribute to freezing of gait in narrow spaces. Thirteen PD participants with freezing (PD FOG), 14 PD participants without freezing (PD non-FOG), and 15 healthy individuals made a perceptual estimate of the width of the distal opening of a corridor in two conditions: parallel and narrowing walls. Gait characteristics and number of freezing episodes were then compared while participants walked in baseline (no corridor), and through parallel walls and narrowing walls corridors. Visuospatial abilities were also assessed using neuropsychological tests. PD FOG had lower scores in the copy of the pentagons (*p* = 0.044) and had greater error variability in the perceptual judgment task (*p* = 0.008) than healthy participants. Although a similar number of freezing episodes occurred in both corridor conditions, PD FOG had greater step length variability while walking through the parallel walls corridor compared to healthy (*p* < 0.001) and PD non-FOG (*p* = 0.017) participants. Regression analysis revealed that error variability in perceptual judgment predicted the percentage of time spent in double support (*R*^2^ = 0.347) only in the narrowing walls condition for PD FOG. These results support the notion that sensory–perceptual deficits both prior to movement planning and during movement execution are important factors contributing to freezing of gait.

## Introduction

One of the most common triggers of freezing of gait (FOG) in Parkinson’s disease (PD) is walking through narrow spaces [1]. In experimental settings, FOG episodes are preceded by a drastic reduction in gait speed and an increase in step-to-step variability while approaching narrow spaces [2, 3], yet it remains unclear how an impaired perception of the environment at early stages of movement planning, or an impaired ability to update and integrate sensory information while interacting with narrow or cluttered spaces might influence the motor planning to successfully pass through the narrow space [4].

Perceptual judgment studies suggest that individuals who experience FOG (PD FOG) are able to judge whether their body can fit through an aperture similarly to both healthy individuals [5] and PD participants who do not experience FOG (PD non-FOG) [3, 6]. Yet, a recent study showed that PD FOG made significantly more judgment errors during a distance estimation task compared to PD non-FOG [7]. In addition, Nantel et al. [8] report visuospatial deficits in PD FOG on cognitive tests that were correlated with objective measures of FOG. Thus, while assessing passability through an aperture is focused on judgments relative to body size, cognitive tests may be too far removed from the reality of passing through a doorway. In this context, it may be valuable to utilize more specific measures of judging aperture width to understand if impaired perception prior to movement contributes to FOG.

A potentially independent issue may be that individuals who experience FOG could be impaired in their ability to update and integrate sensory feedback while walking through a narrow space [2, 3, 9]. In support of this hypothesis, recent neuroimaging studies have shown that individuals with PD experiencing FOG have decreased brain activity in areas involved in sensory integration while imagining themselves walking [10, 11]. More specifically, van der Hoorn and colleagues [12, 13] used an optic flow illusion to assess brain activation involved in perception of forward motion in wide and narrow visual fields. These studies showed that in healthy individuals, a gradual transition from a wide to a narrow visual field resulted in a shift from occipito-parietal and lateral pre-motor area activation to medial pre-frontal areas including the (pre-) supplementary motor area (SMA). They concluded that the transition from a wide to a narrow visual field decreases the visual feedback needed to externally generate a perception of forward motion, and hence activation of areas related to internally generated movement becomes necessary to sustain the intended action. Interestingly, individuals with PD, and to greater extent individuals who experience FOG, neither showed the occipito-parietal activation when a wide visual field was available, nor the activation in pre-frontal medial areas during the gradual transition to a narrow visual field. Thus, FOG in narrow spaces may be associated with an impaired ability to internally generate movement. These previous studies would suggest that moving toward a narrowing space or doorway requires greater internal guidance, and hence a shift in weighting of sensory feedback from vision to other sensory sources such as proprioception. In the current study, we hypothesized that walking through a uniformly constant narrow space (e.g., narrow tunnel) might demand even greater internal guidance than a narrowing space, and hence result in more gait deficits in individual experiencing FOG.

Therefore, this study aimed to evaluate how perceptual deficits prior to movement, and online feedback processing deficits during walking might be associated with FOG behaviors in narrowing compared to a uniformly narrow corridor in PD FOG.

## Materials and methods

### Participants

Fifteen PD FOG, 17 PD non-FOG and 15 healthy participants (HC) were recruited from the Sun Life Financial Movement Disorders Research and Rehabilitation Centre database at Wilfrid Laurier University between July and August 2011. Sample size was based on previous studies involving similar groups [2, 4–6]. PD participants were assessed in their ON medication state. Exclusion criteria include neurological conditions other than PD, inability to walk 10 m unassisted, diagnosis of dementia, and uncorrected visual impairments. Using a previously established protocol [4], PD participants were assigned into PD FOG or PD non-FOG groups.

### Clinical and neuropsychological assessment

The severity of parkinsonian symptoms was assessed by a movement disorders specialist (QJA) using the motor subsection of the Unified Parkinson’s disease Rating Scale (UPDRS-III). Since previous studies have shown that symptom laterality may play an important role in visuospatial deficits in PD [14], the severity of motor symptoms on each side of the body was evaluated using the sum of tremor, rigidity, and voluntary movement scores from the UPDRS-III for each right and left sides. Participants’ general cognitive status was assessed using the Modified Mini-Mental State Exam (3MS). In addition, since FOG has been previously associated with impairments in set shifting [15], this ability was assessed using the Trail Making Test (TMT). Visuospatial processing was evaluated using the copy of the two intersecting pentagons from the 3MS and a line bisection test. In the line bisection task, participants were asked to cross the center of a horizontal line with a pencil. Participants performed eight trials of this task with two line sizes (15 or 20 cm) presented in randomized order (4 lines per size).

### Apparatus

**Fig. 1:**
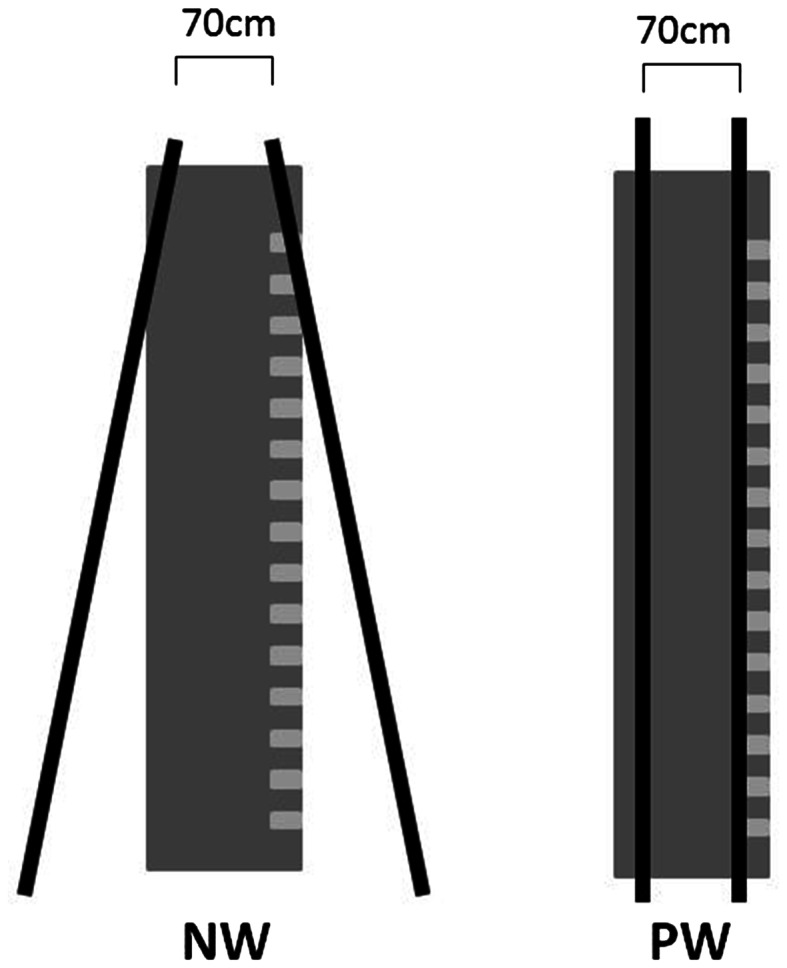
Graphic representation of the experimental conditions. On the *left* *side*, the corridor with narrowing walls (NW) and, on the *right side*, the consistently narrow corridor (PW)

A 2.44-m tall by 7.32-m long corridor was built from wooden material (see Fig. 1). Six 2.44 m height by 2.44 m length movable walls were connected to compose each side of the corridor (three walls on each side) and allowed changing the configuration of the corridor (parallel or narrowing) by sliding the walls over the floor. The interior surface of the walls was built with white plywood and created a homogeneous visual field.

### Perceptual judgment

Participants were sitting 10 m away from the end of a corridor and used an unmarked tape measure to estimate the width of the distal opening of the corridor in two conditions: parallel (PW) and narrowing (NW) walls (Fig. 1). To complete the estimate, participants were required to hold the unmarked tape measure in their own hands out in front of them, so as to judge the width of the opening relative to their hands. The actual width of the aperture that participants were asked to estimate was always the same (70 cm), regardless of the corridor condition. The size of the aperture was chosen based on previous research [2], where the largest effects on gait were found in the narrowest doorway condition that was 0.675 m wide. Participants were instructed to stretch out the tape until they felt that they had an accurate estimate of the distal opening. Then the experimenter locked the tape measure at the point selected by the subject, and took the estimated value in centimeters from the opposite side of the tape which contained the scale. Three trials in each corridor condition were performed in randomized order. Variables of interest were absolute error (AE), constant error (CE), variability of absolute error (VAE). Absolute error was obtained by subtracting the absolute value of participant’s estimate from the value of the aperture size (70 cm). The same calculation was used for constant error, but taking into consideration negative (underestimation) and positive (overestimation) values. To obtain the variability measures, AE standard deviation within-participant was calculated.

### Gait assessment

Participants were instructed to walk at self-selected pace in three conditions: baseline (no corridor), PW, and NW. Participants started within the corridor walls to avoid the transition from an open space to the corridors and were asked to continue walking for approximately 1 m after exiting the corridor. In addition, the first and the last steps of each walking trial were excluded to minimize the effects of acceleration and deceleration on gait parameters. Fifteen trials were performed in blocks of five trials each, starting with baseline. The presentation of the corridor conditions was randomized across participants. Gait parameters were recorded using GAITRite^®^ (26 feet). Gait speed, cadence, step length, step time, step length and step time coefficient of variation (CV), and percentage of time spent in double support were calculated using GAITRite^®^ 4.7 software. To evaluate how movement planning was influenced by the corridor conditions as participants approached the end point of the walkway, step length, step time, their respective CV, and gait speed were also separately assessed in the early (three initial steps), intermediate (three intermediate steps), and late (three last steps) phases of gait task. The number of freezing episodes was counted per condition using video analysis [16].

### Statistical analysis

One-way analysis of variance (ANOVA) and independent *t* tests were used to compare demographic and clinical differences between groups. In the perceptual judgment test, absolute and constant errors were analyzed using a three-factor repeated measures (RM) ANOVA (3 groups × 2 conditions × 3 trials). Error variability was analyzed using a two-factor RM ANOVA (3 groups x 2 conditions). Gait parameters were analyzed using a two-factor RM ANOVA (3 groups × 3 conditions). Since one participant in the healthy group and three participants in the PD FOG group completed only three trials for each of the conditions, the mean value of the walking trials was used for the analysis of overall gait. Including these participants served as an important representation of the difficulties associated with the conditions. Gait behavior in the early, intermediate, and late phases of the gait task was evaluated using a three-factor RM ANOVA (3 groups × 2 conditions × 3 phases). Since it was important to consider how the first encounter with the corridors influenced participant’s movement planning, gait behavior was also evaluated in the first trial. Stepwise regression analysis was used to evaluate whether perceptual judgement measures predict gait behavior in PD FOG. Tukey’s HSD post hoc examined significant differences and alpha level was *p* < 0.05.

### Participants excluded

Three participants in the PD non-FOG group and two participants from the PD FOG group were excluded from the analyses due to inability to complete the perceptual judgment task and poor quality of gait data, respectively. Thus, the following results were based on the analysis of 42 participants (14 PD non-FOG, 13 PD FOG, and 15 HC). After exclusion, PD FOG and PD non-FOG groups remained matched for disease severity. Nevertheless, PD FOG participants had longer disease duration and lower scores in the 3MS than PD non-FOG (Table 1).

**Table 1:** Mean and standard deviation values for clinical and demographic sample characteristics

Group	Age (years)	Sex (M/F)	DD (years)	UPDRS-III	Side affected (R/L)	3MS
HC (*n* = 15)	73.06 (6.69)	9/6	–	–	–	97.47 (2.74)
PD non-FOG (*n* = 14)	68.78 (9.59)	10/4	5 (4.81)	28.14 (8.69)	8/6	96.36 (4.41)
PD FOG (*n* = 13)	74.0 (5.27)	12/1	10.15 (6.47)^b^	31.76 (9.90)	4/9	88.38 (11.24)^a,b^

*DD* disease duration based on years since diagnosis, *UPDRS-III* Unified Parkinson’s disease rating scale motor subsection, *Side affected* number of individuals with most right or left affected body side based on the sum of tremor, rigidity, and voluntary movement scores from the UPDRS-III, *3MS* Modified Mini-Mental State Exam

All significant differences were *p* < 0.05

Group differences: ^a^ HC × PD FOG, ^b ^PD non-FOG × PD FOG (*p* < 0.05)

## Results

### Neuropsychological assessment

The results from neuropsychological tests showed that, at baseline, PD FOG had worse visuospatial functioning than the other two groups. Differences between groups were found in pentagon copying (*F*_(2,39)_ = 3.91; *p* = 0.028), where PD FOG had poorer performance than HC (*p* = 0.044) and tended to have worse performance than PD non-FOG (*p* = 0.054). However, no differences between groups were found in the line bisection task. In addition, while we expected differences in visuospatial function between the FOG patients with left and right side dominance, no differences were identified.

Differences between groups in the TMT were found only for the TMT-B condition (*F*_(2,38)_ = 3.77; *p* = 0.032), where PD FOG participants were slower than HC (*p* = 0.025).

### Perceptual judgment

A main effect of corridor condition for absolute error showed that all participants had more error in their estimate while judging the width of the distal opening of the PW corridor (AE: *F*_(1,39)_ = 4.23; *p* = 0.046). In addition, a main effect of corridor condition for constant error showed that all participants underestimated the size of the corridor PW (CE: *F*_(1,39)_ = 14.66; *p* < 0.001).

A main effect of group was found for error variability (*F*_(2,39)_ = 4.99; *p* = 0.011), where PD FOG participants were more variable than HC (*p* = 0.008) across both corridor conditions. Similar to the baseline neuropsychological testing for visuospatial function, no differences between left and right dominant PD FOG were identified.

### Overall gait behavior

The following section describes all interactions found in the current study, while main effects of group and corridor conditions are found in Table 2.

**Table 2:** Effects of group and corridor condition on gait characteristics

	Baseline	PW corridor	NW corridor	Group effects	Corridor effect	Group × corridor
Gait speed (cm/s)				*p* < 0.0001^a,b^	*p* < 0.001^a,b^	*p* = 0.069
HC	120.93 (19.89)	128.22 (20.88)	128.78 (21.20)			
PD non-FOG	102.40 (19.99)	106.18 (24.93)	108.10 (20.93)			
PD FOG	86.71 (20.03)	85.08 (21.59)	88.31 (24.10)			
Cadence (step/min)				*p* = 0.78	*p* < 0.0001^a,b^	*p* = 0.96
HC	107.48 (10.59)	111.02 (10.92)	111.25 (10.75)			
PD non-FOG	104.72 (10.24)	108.95 (11.42)	108.03 (10.32)			
PD FOG	104.89 (11.91)	108.67 (15.27)	108.27 (16.84)			
Step length (cm)				*p* < 0.0001^a,b,c^	*p* = 0.02^c^	*p* = 0.012
HC	67.54 (9.85)	69.42 (10.54)	69.58 (10.47)			
PD non-FOG	58.26 (8.04)	57.90 (9.87)	59.73 (8.68)			
PD FOG	49.86 (10.62)	47.49 (11.63)	49.47 (12.46)			
Step length CV (%)				*p* = 0.0001^b,c^	*p* < 0.0001^a,c^	*p* = 0.001
HC	3.15 (1.06)	3.2 (0.92)	3.05 (0.75)			
PD non-FOG	4.44 (2.12)	5.48 (3.72)	5.10 (2.74)			
PD FOG	6.71 (3.39)	9.62 (5.24)	7.73 (3.96)			
Step time (s)				*p* = 0.71	*p* < 0.0001^a,b^	*p* = 0.91
HC	0.56 (0.056)	0.54 (0.055)	0.54 (0.054)			
PD non-FOG	0.57 (0.055)	0.55 (0.059)	0.56 (0.054)			
PD FOG	0.57 (0.071)	0.56 (0.083)	0.56 (0.085)			
Step time CV (%)				*p* = 0.0005^b,c^	*p* = 0.051	*p* = 0.073
HC	3.28 (1.28)	3.03 (1.24)	2.83 (1.32)			
PD non-FOG	4.61 (2.55)	4.68 (2.42)	3.90 (1.48)			
PD FOG	6.09 (3.00)	7.83 (4.47)	6.67 (3.92)			

Group differences: ^a^ HC × PD non-FOG, ^b^ HC × PD FOG, ^c^ PD non-FOG × PD FOG

Corridor condition differences: ^a^ baseline × PW, ^b^ baseline × NW, ^c^ PW × NW (*p* < 0.05)

**Fig. 2:**
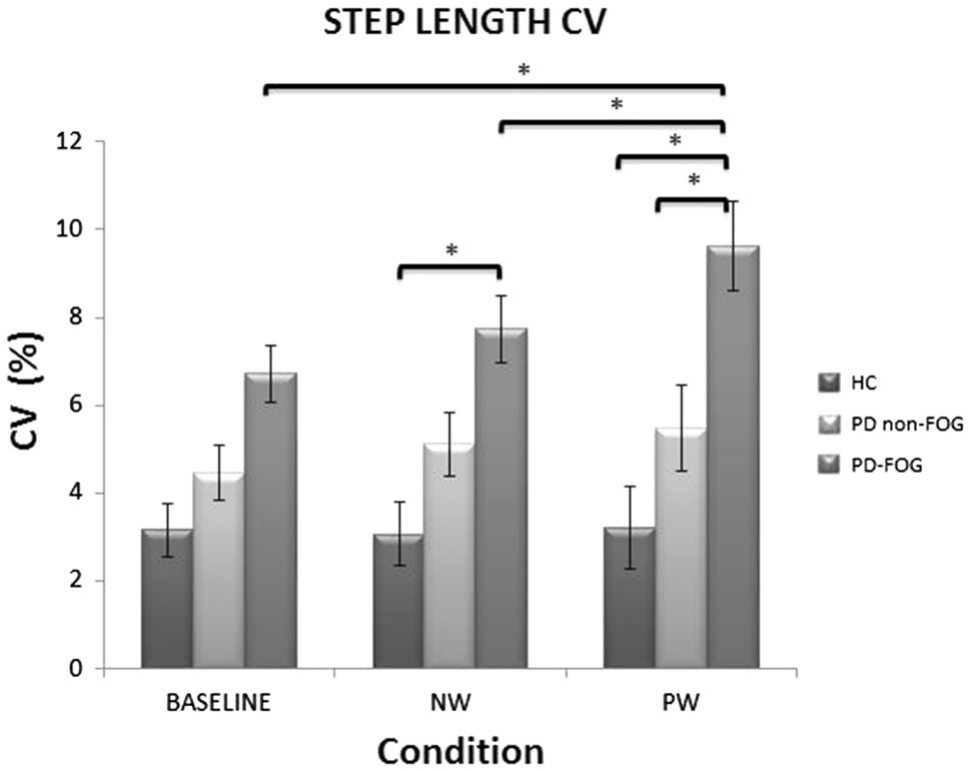
PD FOG participants had greater step length variability while walking through the continuously narrow corridor (PW) compared to healthy and PD non-FOG participants

Group by corridor interactions were found for both step length (*F*_(4,78)_ = 3.39; *p* = 0.012) and step length variability (*F*_(4,78)_ = 4.81; *p* = 0.001). However, since the post hoc tests for step length did not reveal clear differences to support the interaction, we focused on the step length variability interaction. For step length variability (Fig. 2), PD FOG had greater variability in the PW corridor compared to PD non-FOG (*p* = 0.017) and HC (*p* < 0.001). In the NW corridor, the PD FOG group only differed from the HC group (*p* = 0.003). PD non-FOG participants were not different than HC in any condition.

A group by corridor condition interaction was found for the percentage of double support time (*F*_(4,78)_ = 6.59; *p* = 0.0001), where only the PD FOG group increased the percentage of time spent in double support in the PW corridor compared to baseline (*p* = 0.017).

### Gait behavior in walking phases

An interaction between group and phase for step length (*F*_(4,78)_ = 4.36; *p* = 0.003) demonstrated that the HC group walked with similar step length through all phases of gait task, whereas both PD groups walked with smaller steps in the late phase (*p* < 0.001) regardless of condition. For step length variability, it was found that all participants walked with greater variability in the late phase (*F*_(2,78)_ = 8.50; *p* < 0.001) in both corridor conditions.

Although there were no significant results for step time, an interaction between group and phase was found for step time variability (*F*_(4,78)_ = 2.63; *p* = 0.04), where only PD FOG had greater step time variability in the late compared to the early (*p* = 0.02) and intermediate (*p* = 0.03) phases. An interaction between group, corridor condition, and phase was found for step time variability in the first trial (*F*_(4,78)_ = 3.72; *p* = 0.007). This interaction showed that in the first time PD FOG walked through the NW corridor, step time variability increased in the late phase (*p* = 0.005), while no changes between phases were found in the PD non-FOG or HC groups. Interestingly, step time variability was similar in all phases and for all groups in the corridor PW in the first trial.

An interaction between group and walking phase (*F*_(4,78)_ = 3.00; *p* = 0.023) showed that in both corridors, all participants with PD had slower speed in the late compared to other phases (*p* < 0.001), while the late phase was only different than the intermediate phase for HC (*p* = 0.002).

### Number of freezing episodes

FOG episodes occurred at 1 % of trials in the baseline condition, 5 % in the PW condition, and 5 % in the NW condition.

### Relationship between visuospatial processing and gait behavior in FOG

**Fig. 3:**
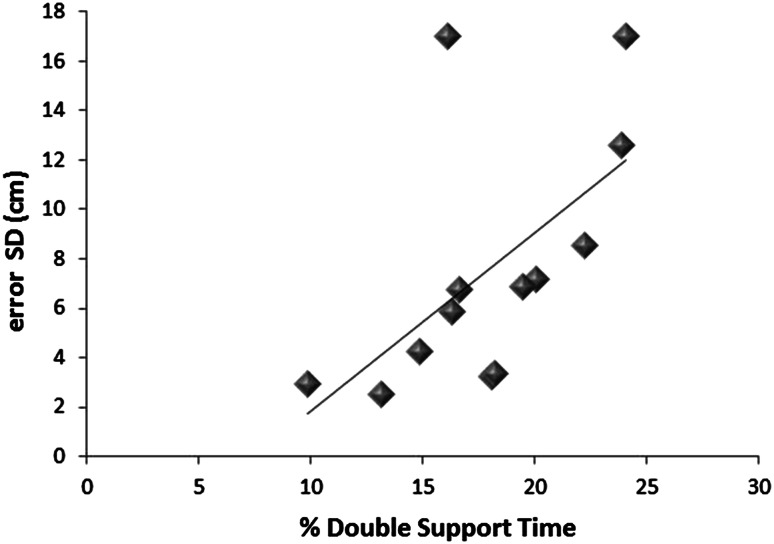
Greater error variability in the perceptual judgment task was associated with the percentage of time spent in double support only for PD FOG participants and only in the NW condition

Regression analysis revealed that the variability of absolute error in the perceptual judgment predicted the percentage of time spent in double support in the PD FOG group exclusively in the NW corridor (*R*^2^ = 0.347) (Fig. 3).

## Discussion

This study aimed to investigate whether visuospatial processing (necessary for planning prior to movement) and sensory updating contribute to FOG in narrow spaces. Further, we wanted to verify if visuospatial abilities were correlated with gait behavior of PD FOG participants while walking through narrow spaces. Interestingly, PD FOG performed worse on visuospatial processing tests compared to healthy participants and showed a tendency for worse performance than PD non-FOG. Moreover, PD FOG perceptual judgments of aperture width were more variable than those of healthy participants. Importantly, only the PD FOG group was differentially influenced by the narrow corridor conditions during the walking trials. PD FOG walked with greater step length variability and spent more time in double support while walking through PW corridor compared to both NW corridor and baseline, whereas PD non-FOG and healthy participants showed no differences in these measures across all three conditions. Careful consideration of the relationship between perceptual judgment prior to movement onset, and how walking performance is influenced by narrow spaces is critical to our understanding of FOG behaviors, and so the results of these experiments are discussed in greater detail below.

The results from neuropsychological tests support previous findings [7, 8] that PD FOG participants do in fact display visuospatial deficits. Interestingly though, while absolute and constant error of aperture width estimations did not differ between groups, PD FOG participants were more variable in their judgment than healthy participants. This finding is in contrast with the results from Cowie et al. [5], who did not find differences in error variability when comparing PD FOG to healthy participants. However, in this previous study, the perceptual judgment task was performed after walking trials and thus patients had the experience of passing through the opening prior to making any judgments. The previous study also involved individuals’ ability to judge whether they could pass through different aperture widths, which may be more focused on evaluating a crude sense of body scaling relative to the environmental surroundings. Given that the current study required a more specific assessment of aperture width (independent of body size), and since the perceptual judgment task was completed prior to walking through the narrow spaces, it is likely that the current results provide a closer look at perceptual impairments while avoiding any biases associated with the experience of having already passed through the narrow spaces.

With visuospatial deficits established in the PD FOG group prior to initiation of movement, the second objective was to evaluate how different conditions of narrowness might influence gait and freezing behaviors. Based on a recent protocol that aimed to evaluate how planning might be reflected in goal-directed tasks [17], the entire walk was divided into three phases (early, middle and late). PD FOG showed increased step time variability when first walking through the NW corridor, which dramatically increased in the late phase (narrowest point). Therefore, since step time variability was highest at the narrowest point of the NW corridor in PD FOG, this might suggest that controlling gait to safely pass through the aperture requires the greatest processing demands, as has been previously suggested with increases in step time variability [18].

Interestingly, the PD FOG group was more influenced by the PW corridor than the NW corridor, as seen with both increased step length variability and increased percentage of time spent in double support. In the present study, a consistently narrow space (PW) likely required more internally generated movement and, as a result, a shift in weighting of sensory feedback from visual to other sensory sources such as proprioception. A recent study demonstrated that when PD FOG relied on proprioceptive feedback to avoid the threat of collision imposed by a doorway, the number of freezing episodes increased significantly. The authors suggested that the integration of proprioceptive feedback may be a core factor that underlies FOG [9]. In the current study, the PW corridor likely involved heightened utilization of proprioceptive feedback to improve body awareness relative to the corridor walls, to avoid any contact with the walls. This conclusion would also serve as an alternative explanation for the increased gait variability in only the late phase of the NW condition.

Finally, it was important to consider whether visuospatial processing deficits could be associated with changes in gait behavior in PD FOG individuals. With multiple regression analysis, error variability in the perceptual judgment task was the only variable that predicted percentage of time spent in double support, and specifically in the NW corridor. One possible explanation may be that increased variability in perceptual judgment in PD FOG results in longer double support time to better sample and integrate sensory feedback into the planned movement, in compensation for the uncertainty (heightened variability) in perceiving the environment. Previous research has suggested that increased time spent in double support is an objective measure of freezing behavior [3]. However, it is difficult to know whether increased percentage of time spent in double support in this study was a strategy used by PD FOG participants to enhance processing of sensory feedback or whether the uncertainty of the size of the aperture resulted in a behavior that could lead to a freezing episode. Thus, it is suggested that the need to frequently update changes in the environment in the NW condition (to assess when the narrowest point will be encountered) necessitates the increased double support time and increased gait variability found in the study.

Although several studies have provided consistent evidence of a relationship between FOG and cognition [15, 19–24], it is important to note that of the cognitive measures employed in the current study, correlational analyses revealed that there were no associations with gait behavior in PD FOG. Thus, findings in this study do not appear to be the result of general cognitive status (3MS) or set-shifting ability (TMT) in PD FOG. Nonetheless, it is important to consider alternatives that might explain these results. For example, we might have expected that symptom laterality might influence these results, especially given the fact that we had more freezers with left side as the predominant side affected. However, the comparison between right and left PD patients did not reveal a laterality effect for the baseline visuospatial tests, nor the perceptual judgement tests. Thus, gait behavior differences between groups cannot be attributed to visuospatial deficits in this study. Another example, emotional state was not assessed in this study, but perhaps anxiety should also be evaluated in future studies.

In summary, this study provides evidence of visuospatial deficits prior to movement in PD FOG, which likely influences gait control while attempting to update self-motion information in approaching a narrow space. Thus, freezing behaviors may result from circumstances where processing demands are increased as a result of the mismatch between visuospatial judgment and sensory feedback needed to correct it. Considering this relationship may be key to our understanding of the elusive freezing phenomenon.
